# 18F-Choline PET/CT or PET/MR and the evaluation of response to systemic therapy in prostate cancer: are we ready?

**DOI:** 10.1007/s40336-022-00515-7

**Published:** 2022-07-28

**Authors:** Luca Urso, Federica Lancia, Naima Ortolan, Marta Frapoli, Martina Rauso, Paolo Artioli, Corrado Cittanti, Licia Uccelli, Antonio Frassoldati, Laura Evangelista, Mirco Bartolomei

**Affiliations:** 1 Department of Translational Medicine, University of Ferrara, Ferrara, Italy; 2 Nuclear Medicine Unit, Oncological Medical and Specialists Department, University Hospital of Ferrara, Via Aldo Moro 8, 44124 Ferrara, Italy; 3 Oncological Medical and Specialists Department, Oncology Unit, University Hospital of Ferrara, Ferrara, Italy; 4 Nuclear Medicine Unit, Department of Medicine, DIMED University of Padua, Padua, Italy

**Keywords:** Prostate cancer, PET, Choline PET, Therapy response assessment

## Abstract

**Purpose:**

During the last decade, [18F]F-choline positron emission tomography (PET) had a rising role in prostate cancer (PCa) imaging. However, despite auspicious premises, [18F]F-choline PET is not currently recommended for the evaluation of response to therapy assessment in PCa, mainly due to the lack of large-scale prospective trials.

**Methods:**

We report the cases of seven patients affected by PCa, in which [18F]F-choline PET (either with computed tomography—CT or magnetic resonance imaging—MR) contributed significantly in the systemic therapy response evaluation.

**Results and conclusion:**

[18F]F-choline PET/CT or PET/MR demonstrated to be a useful imaging modality in the assessment of response to systemic therapy in metastatic PCa patients, irrespective of the stage of disease (either in hormone sensitive and in castrate resistant condition) and the kind of systemic treatment. In most cases, PSA serum values and [18F]F-choline PET showed a synchronous disease evolution after systemic therapy. ADT can alter [18F]F-choline uptake, therefore the time of scan should be correctly planned. Finally, PET/CT with [18F]F-choline is a useful tool for reinforcing the identification of metastatic disease in case of a switch from metastatic castration sensitive to castration resistant PCa.

## Introduction

During the last decade, [18F]F-choline positron emission tomography (PET) had a rising role in prostate cancer (PCa) imaging. Choline is a component of phosphatidylcholine, one of the main component of cell membranes [1]. Although initially interpreted as a direct sign of high cell proliferation rate, recent biochemical evidences correlated increased choline metabolism—in particular upregulation of choline kinase—with malignant transformation in cancer cells, especially in PCa [2]. Currently, [18F]F-choline PET is indicated for recurrent PCa, mainly in case of PSA elevation during follow-up [3, 4].

Therapy response is currently evaluated by analysing several factors including: modifications of serum prostate-specific antigen (PSA) levels, Response Evaluation Criteria in Solid Tumors (RECIST 1.1) on serial contrast enhanced computed tomography (ceCT) [5], and bone scan. However, changes in PSA values are often inconsistent during therapy [6]. Also, RECIST 1.1 criteria are not reproducible in PCa patients, due to different factors, such as the measurement of bone lesions, the heterogeneous response among the different lesions and for the appearance of pseudo-progression phenomena [6, 7]. Bone scan has also several limitations, in particular a low specificity, and the inability to distinguishing between a true progression and a flare phenomenon [6].

In this scenario, clinicians need a reliable and standardized imaging method, to better assess therapy response, similarly to what happens in other neoplasms [8, 9]. Despite auspicious premises, [18F]F-choline PET is not currently recommended for the evaluation of response to therapy assessment in PCa, mainly due to the lack of large-scale prospective trials [7, 10, 11]. Indeed, although several literature evidences have been published, most of those data were obtained from retrospective analyses of small cohorts of patients [1, 3, 6, 7, 10, 12–15]. Similarly, PSMA-ligands PET imaging presents the same limitations in the therapy response assessment, both on PCa and other kind of neoplasms [11, 16].

The aim of this pictorial essay was to describe some cases of PCa patients who underwent [18F]F-choline PET for the evaluation of response to different systemic treatments, providing some considerations in accordance with the current literature data.

## Materials and methods

We retrospectively evaluated seven PCa patients (aged 64–83 years) in several different lines of treatment for metastatic disease. Patients performed [18F]F-choline PET between January 2017 and April 2022 in two different Italian Nuclear Medicine Units University of Ferrara (Center A) and University of Padova (Center B). Among selected patients, two had castration sensitive prostate cancer (CSPCa) while the remaining five had castration resistant prostate cancer (CRPCa). Disease was monitored with serial serum PSA samples, performed close to the [18F]F-choline PET scans.

### PET/CT image protocol and interpretation (Centre A)

Images were acquired from the mid-thigh to the skull vertex about 60 min after [18F]F-choline injection (3 MBq/Kg) using a standard technique on a dedicated PET/CT system (Biograph mCT Flow; Siemens Medical Solutions, Malvern, PA, USA). After non-contrast-enhanced low-dose CT (120 keV, 80 mAs, CareDose; reconstructed with a soft-tissue kernel to a slice thickness of 3 mm), PET was acquired in 3-dimensional mode (matrix, 200 • 200) using FlowMotion (Siemens). The emission data were corrected for randoms, scatter, and decay. Reconstruction was performed with Syngo Acquisition Workplace; attenuation correction was performed using the non-enhanced low-dose CT data. All images were processed and analysed on a Syngo.via Workstation (Siemens Healthineers) by two experienced nuclear medicine physicians.

### PET/MR image protocol and interpretation (Centre B)

PET/magnetic resonance MR were acquired using hybrid equipment (Biograph mMR®; Siemens Healthcare, Erlangen, Germany) with 3 T MR. [18F]F-choline was administered intravenously (at a dose of 3 MBq/kg of body weight). PET study of the pelvis was conducted for 30 min while simultaneously acquiring T2-weighted turbo spin-echo, T1-vibe after contrast enhancement, T2-haste, T1-vibe fat-saturated, and diffusion-weighted (DWI b50, 800 and 1400) sequences for the MR component. Then total-body PET images were acquired using a 3 min-per-bed protocol with the simultaneous acquisition of T2-haste, T1-vibe fat-saturated (3 mm thick), and DWI sequences (b50 and 1000). A T1-vibe sequence (2 mm thick) was used for the lung scan. MR based attenuation correction maps (MRAC) were calculated using a 4-class segmentation technique [17]. All images were reviewed by two nuclear medicine physicians and two radiologists with at least 10 years of experience with PET and MR, respectively, using dedicated software (Syngo.via, Siemens Healthcare, Erlangen, Germany). PET/MR scans were interpreted as follows: PET was defined as positive in the presence of an [18F]F-choline uptake greater than that of the background activity, excluding foci of physiological activity. MR was considered positive in the case of an increased enhancement after administering the contrast agent and subsequent rapid wash-out in the MR sequences, or a significantly restricted diffusion in the DWI sequences. The RECIST 1.1 criteria were used to interpret the MR, in particular for lymph node and bone recurrences. PET/MR was considered positive in patients found positive on PET or MR, or both.

## Results

### Hormone sensitive vs castration resistant prostate cancer: Case 1 and Case 2

**Fig. 1:**
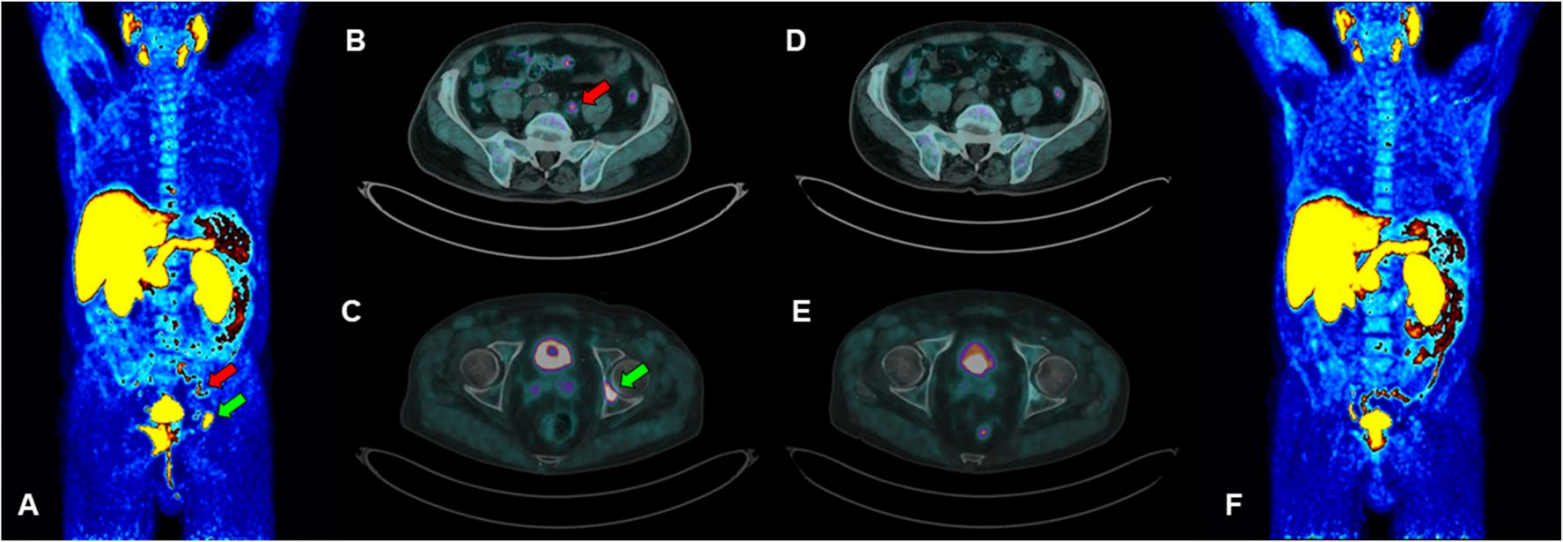
Staging [18F]F-choline PET/CT scan (**A** maximum intensity projection; **B**–**C** transaxial fused images): the exam shows skeletal (green arrows) and lymph node (red arrows) uptakes. A second [18F]F-choline PET/CT scan performed 10 days after the start of ADT (**D**–**E** transaxial fused images; **F** maximum intensity projection) showed almost complete disappearance of pathological uptakes (i.e. SUVmax dicreasing from 33.2 to 3.4 on acetabulum lesion)

CASE 1. A 74 year-old man with a new diagnosis of PCa (Gleason Score—GS—equal to 9, 4 + 5; ISUP grade: 4) and a serum PSA value of 2.82 ng/ml, underwent [18F]F-choline PET/CT for the initial staging of disease. A pathological tracer uptake was reported in the prostate gland, multiple abdominal lymph nodes, bone (acetabulum and some vertebral metameres), and two pulmonary lesions (Fig. 1 A–C). Ten days after the start of androgen deprivation therapy (ADT), PSA value was 1.67 ng/ml and a second [18F]F-choline PET/CT was performed showing only a minimal and blurred uptake in correspondence of the skeleton (Fig. 1 D–F). In the following months, PSA values were always lower than 0.50 ng/ml, with suppressed testosterone values. ADT is still ongoing.

This case is consistent with literature evidence, which report that ADT reduces [18F]F-choline uptake in PCa, particularly in hormone-naïve patients. Indeed, androgens are necessary for PCa cells’ growth; therefore, depriving the organism of androgens with ADT induced glandular atrophy and apoptosis [18] and the [18F]F-choline uptake is reduced. What makes this case unique is the timing between the start of the ADT and the performance of [18F]F-choline PET/CT. The patient started ADT 10 days before performing [18F]F-choline PET/CT, but an almost complete response was already found. We may therefore speculate that [18F]F-choline PET/CT is a valuable tool to early assess the response to therapy in hormone sensitive PCa, although a slight effect on the PSA level. One more teaching point is that patients who undergo [18F]F-choline PET/CT for staging PCa should perform it before starting ADT. Otherwise, even if ADT was started a few days before the scan, false negative findings have to be expected, hesitating in a down-staging of patient’s disease.

**Fig. 2:**
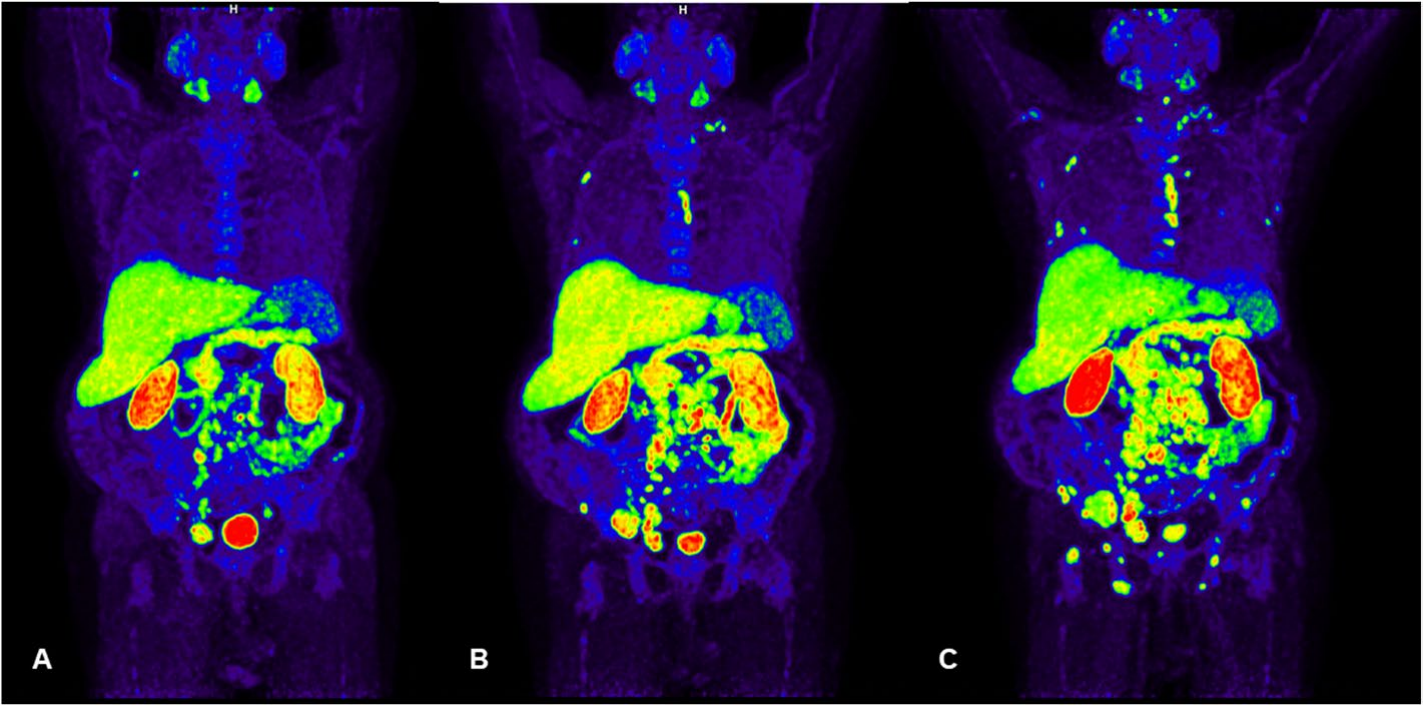
Baseline [18F]F-choline maximum intensity projection image (**A**) showed multiple skeletal and lymph node uptakes. Restaging [18F]F-choline maximum intensity projection images, performed 9 (**B**) and 11 (**C**) months after starting ADT respectively, showed a progressive increase of the bone and lymph node pathological uptakes (i.e. SUVmax progressively raising from 11.4 to 20.0 to 31.1 on L3 bone lesion)

CASE 2. A 68 year-old man with a baseline PSA level of 71.29 ng/ml. Due to the COVID-19 pandemic emergency, the patient repeated the PSA assay 8 months later, with a marked increase in its value (264 ng/ml). Consequently, multiparametric-MR and [18F]F-choline PET/CT were performed, with evidence of a Pi-RADS-5 prostate lesion, multiple lymph node and bone metastases (Fig. 2A). Fusion guided prostate biopsy confirmed the diagnosis of PCa (GS 6 (3 + 3) ISUP grade: 1) and, therefore, ADT was started, achieving a rapid decrease of PSA values to 19.65 ng/ml, with suppressed values of testosterone. In the following months, the patient underwent two serial [18F]F-choline PET/CT scans. The last one showed an important progression of the disease at the level of bones and lymph nodes (Fig. 2 B,C), associated with an elevation of the serum PSA level (235 ng/ml). According to both high volume of disease and PSA value, a new line of therapy for CRPCa was started and it is still ongoing (docetaxel plus androgen deprivation therapy).

This case underlines the relevance of [18F]F-choline PET/CT in detecting the switch to CRPCa. Indeed, even though ADT would reduce radiolabelled choline uptakes, [18F]F-choline PET/CT shows true positive findings when the transition to androgen independence occurs [18, 19]. This is consistent with in vitro data [20]. Therefore, [18F]F-choline PET/CT would be a useful tool to restage patients undergoing ADT, in case of suspicious shift to a castrate resistant condition.

### Metabolic response vs clinical response: case 3, case 4 and case 5

**Fig. 3:**
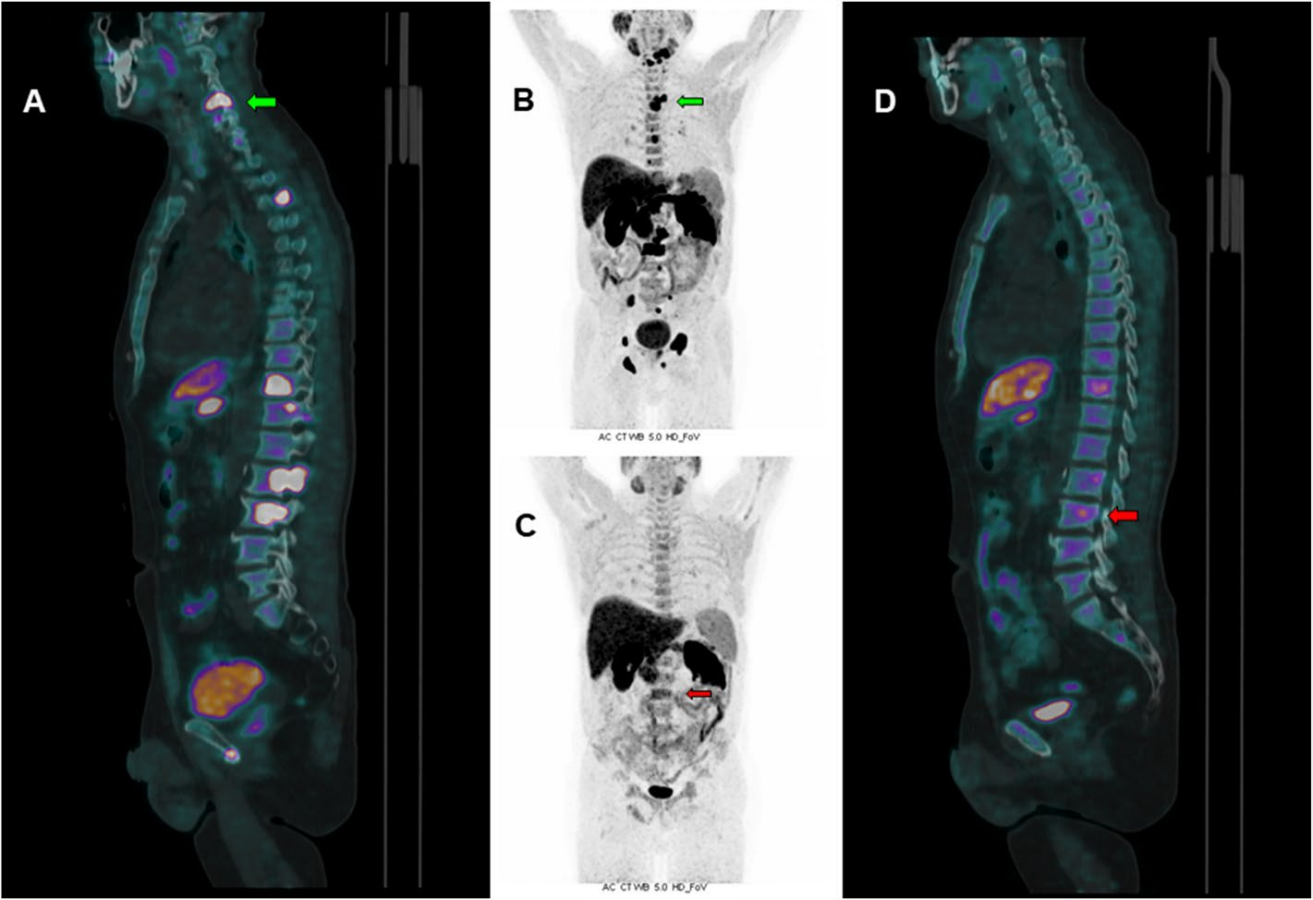
Baseline [18F]F-choline PET/CT scan (**A** sagittal fused image, **B** maximum intensity projection) showed prostatic increased uptake and several bone uptakes (i.e. SUVmax 25.5 on L3), suggestive for metastasis (green arrow). [18F]F-choline PET/CT scan, performed about 7 months later, with ongoing ADT plus docetaxel therapy (**C** maximum intensity projection; **D** sagittal fused image) showed only minimal residual vertebral uptake (red arrow, SUVmax 10.3 on L3)

CASE 3. A 65-year-old man with an increased serum PSA value (25.89 ng/ml) underwent prostate biopsy, with a diagnosis of PCa, GS 7 (4 + 3), ISUP group 3. A baseline [18F]F-choline PET/CT showed an intense tracer uptake in correspondence of the prostate gland, of some para-rectal lymph nodes and of almost all of the examined bone segments (Fig. 3 A,C). Therefore, first-line treatment with docetaxel (75 mg/mq plus prednisone plus androgen deprivation therapy) was started, in accordance with the CHAARTED and STAMPEDE trial [21, 22]. After 6 cycles of therapy, PSA value significantly decreased (0.12 ng/ml); a new [18F]F-choline PET/CT scan showed a complete metabolic response in the majority of skeletal lesions, except for two vertebras (D11 and L3) (Fig. 3 B,D).

**Fig. 4:**
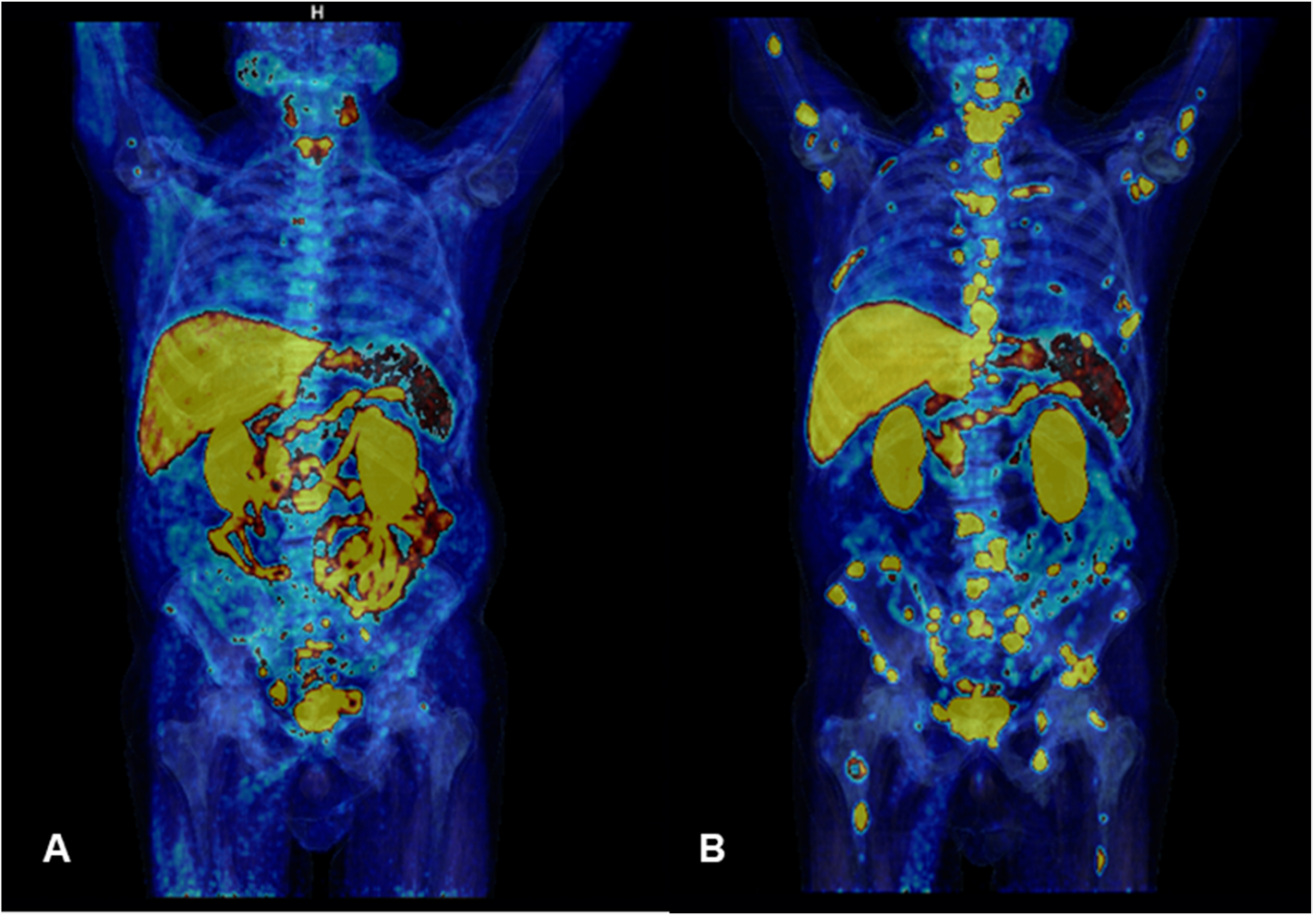
Baseline and post enzalutamide [18F]F-choline maximum intensity projection images (**A** and **B** respectively). Restaging [18F]F-choline PET/CT showed the appearance of multiple skeletal uptakes (i.e. SUVmax 21.3 on sternal body)

CASE 4. A 76 year-old man with diagnosis of PCa, GS 8 (5 + 3) ISUP grade 4, underwent radical prostatectomy and left iliac obturator lymphoadenectomy (pT3 N0 M0). PSA nadir value (0.03 ng/ml) was achieved in 4 months. After 6 months, due to biochemical recurrence, a [18F]F-choline PET/CT was performed showing a focal tracer uptake within the prostate lodge and in multiple abdominal lymph nodes. Over the three following years, the patient received several lines of hormonal therapy. Four years from diagnosis, due to a rapid increase of PSA value (20.76 ng/ml), a new [18F]F-choline PET/CT scan was performed. The exam demonstrated a progression of disease at bone and lymph nodes (Fig. 4A); therefore, a new line of treatment based on enzalutamide was started. After 7 months, serum PSA value (35.56 ng/ml) increased and a further [18F]F-choline PET/CT showed a marked progression of disease (Fig. 4B). In the following months, the patient's clinical condition rapidly deteriorated and he died at the age of 80 years.

These two cases underline the usefulness of [18F]F-choline PET/CT in assessing systemic therapy response to both chemotherapy and second-generation ADT agents in metastatic PCa. Only a few papers analysed the modifications in [18F]F-choline PET/CT induced by chemotherapy [6, 10, 14]. However, being [18F]F-choline tracer directly correlated to the phosphocoline metabolism, it is representative of the number of cells surviving after chemotherapy [10]. Moreover, in patients with decreasing PSA values after chemotherapy, the finding of a metabolic progression at [18F]F-choline PET/CT imaging could represent an alarm for a forthcoming clinical and biochemical disease progression [6]. On the other hand, a few literature evidence evaluated the role of [18F]F-choline PET/CT in the assessment of the response to therapies with second-generation ADT agents [1, 15]. De Giorgi et al. [1] concluded that [18F]F-choline PET/CT in tandem with PSA dosages may give an overall assessment of therapy response in metastatic CRPCa patients treated with enzalutamide. Moreover, Maines et al. [7] reported a prognostic value of a baseline [18F]F-choline PET/CT in patients receiving enzalutamide. Although these two patients had very different settings of disease (CRPCa vs CSPCa and chemotherapy vs second-generation ADT agents) in both cases, [18F]F-choline PET/CT demonstrated to be a useful imaging modality in the assessment of response to several lines of therapy. Serum PSA values and [18F]F-choline PET/CT imaging showed a consistent trend of response for both patients, even though the two clinical histories demonstrated an opposite evolution. More trials are needed to strengthen our knowledge in therapy response assessment using metabolic imaging.

**Fig. 5:**
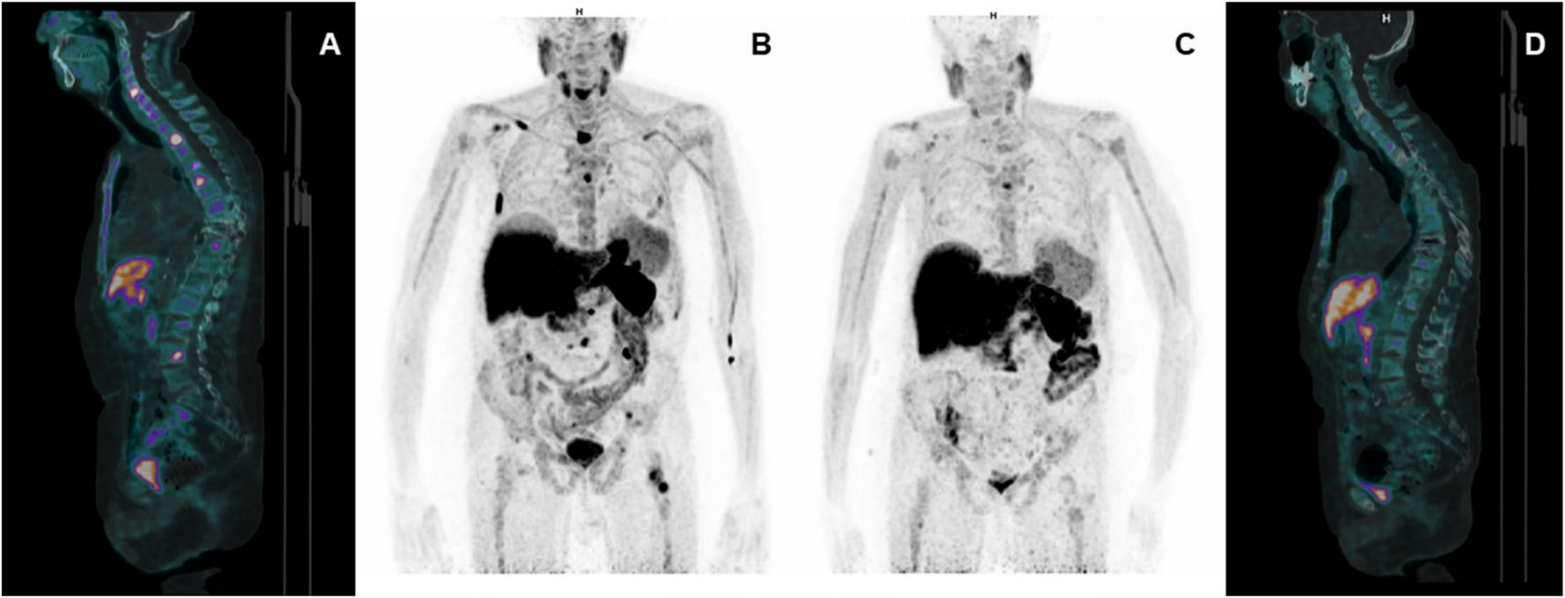
Baseline [18F]F-choline PET/CT scan (**A** sagittal fused image and **B** maximum intensity projection) showed several skeletal PCa pathological uptakes. Post-treatment [18F]F-choline PET/CT scan (**C** maximum intensity projection; **D** sagittal fused image), performed after [223Ra]RaCl_2_ therapy showed a significant reduction of metastatic bone uptakes (i.e. SUVmax decreasing from 19.3 to 5.4 on the left femoral neck lesion)

CASE 5. A 82 year-old man with metastatic PCa (GS 7 (3 + 4), ISUP grade 2) treated with radical prostatectomy, several lines of systemic therapy and salvage radiation therapy was referred to the Nuclear Medicine Unit of the University Hospital of Ferrara to be treated with [223Ra]RaCl_2_ after progression to Enzalutamide. The patient showed several skeletal PCa metastases (Fig. 5 A,B) at baseline [18F]F-choline PET/CT and with an ECOG Performance Status equal to 3. Therapy was performed in accordance to the current European Association of Nuclear Medicine (EANM) guidelines [23]; a progressive, significant reduction of pain associated to skeletal lesions was reported by the patient, after 3 cycles of therapy. No adverse effects occurred. Blood tests showed a moderate progressive reduction of haemoglobin, from 11.4 g/dl at the 1st cycle to 9.1 g/dl at the 6th cycle. Serum PSA values showed a progressive increase from 58 ng/ml to 166 ng/ml at the last cycle. [18F]F-choline PET/CT was performed 45 days before and after [223Ra]RaCl_2_ therapy, demonstrating a significant reduction in the number and for the intensity of uptake in skeletal metastases (Fig. 5 C,D), compatible with a partial metabolic response to therapy, although the increase in PSA levels. Alkaline phosphatase (ALP) was equal to 68 UI/ml after the 1st cycle, 51 UI/ml at the end of [223Ra]RaCl_2_ therapy and 38 UI/ml at the time of last PET/CT scan, therefore a similar trend between choline uptake and ALP was found. Unfortunately, the patient died a few months later for complications unrelated to PCa and it was not possible to dose PSA values again.

This case underlines the possible misleading PSA trend in therapy response assessment, sometimes referable to a flare phenomenon more than to a real progression [23, 24]. In this context, [18F]F-choline PET/CT agreed with ALP reduction and patient’s clinical improvement, displaying a significant response to therapy, even though, a confirmation could not be obtained due to patient death for other reasons.

### Mismatch between PSA and [18F]F-choline uptake: Case 6

CASE 6. A 83 year-old man, with a history of PCa diagnosed 18 years before (pT4 pN0 pMx infiltrating the bladder, GS 6, ISUP grade 1, starting PSA serum values of 4.7 ng/ml), was performing routine follow-up with PSA serum values and [18F]F-choline PET/MR. At diagnosis, the patient was treated with radical prostatectomy, with persistence of dosable PSA values (1.37 ng/ml) but with a negative [18F]F-choline PET/CT. Consequently, the patient underwent prostate bedradiation therapy, with a reduction of PSA serum values to 0.32 ng/ml. As a consequence, follow-up with periodic PSA dosages was established. Five years ago, serum PSA values showed a quick growth (PSA 3.75 ng/ml, PSA doubling time 5.3 month) and [18F]F-choline PET/CT showed a focal uptake in a para-rectal nodule, suggestive for disease relapse. Therefore, LH-RH analogue therapy was started, with a rapid decrease in PSA serum values (0.01 ng/ml after 3 months of treatment). After two years of stable disease, PSA value was still 0.02 ng/mL. However, due to clinical symptoms such as pelvis pain and stipsi, a [18F]F-choline PET/MR (first PET/MR scan) was performed showing the increase in choline uptake in the rectum that correspond to a solid tissue at MR images and the appearance of a new area of focal uptake in correspondence of the right ischium-pubic ramus that was confirmed also at MR images, referable to progressive disease. Thus, the patient started a first-line therapy with Enzalutamide. For the assessment of response to systemic therapy, 8-months later, although stable PSA serum values (0.01 ng/ml), the patient was again submitted to [18F]F-choline PET/MR (second PET/MR scan) that showed a further disease progression (increasing uptake for the lesions already described and a new uptake in the right ala of the sacrum). However, the therapy was continued in the same way. The latest scan, performed in the early February 2022 (third PET/MR scan) with a similar PSA level, showed a further progression of disease (appearance of new bone lesions and liver metastases visible at PET and MR images). Therefore, the patient was submitted to docetaxel-based chemotherapy.

The present case is interesting for the non-dosable PSA level and a positive [18F]F-choline PET/MR. Based on the available data in literature, in case of undifferentiated PCa (high GS), [18F]F-FDG PET/CT would be used [25], due to its ability to detect the metastasis and also for providing prognostic information. In the present case, a significant [18F]F-choline uptake was found, although an undifferentiated PCa. Therefore, also in case of low PSA levels, and aggressive disease (GS higher than 8), a [18F]F-choline PET/CT would be suggested for the staging of metastatic disease and to monitor the response to systemic therapy.

### Visceral progression of disease during systemic therapy: Case 7

**Fig. 6:**
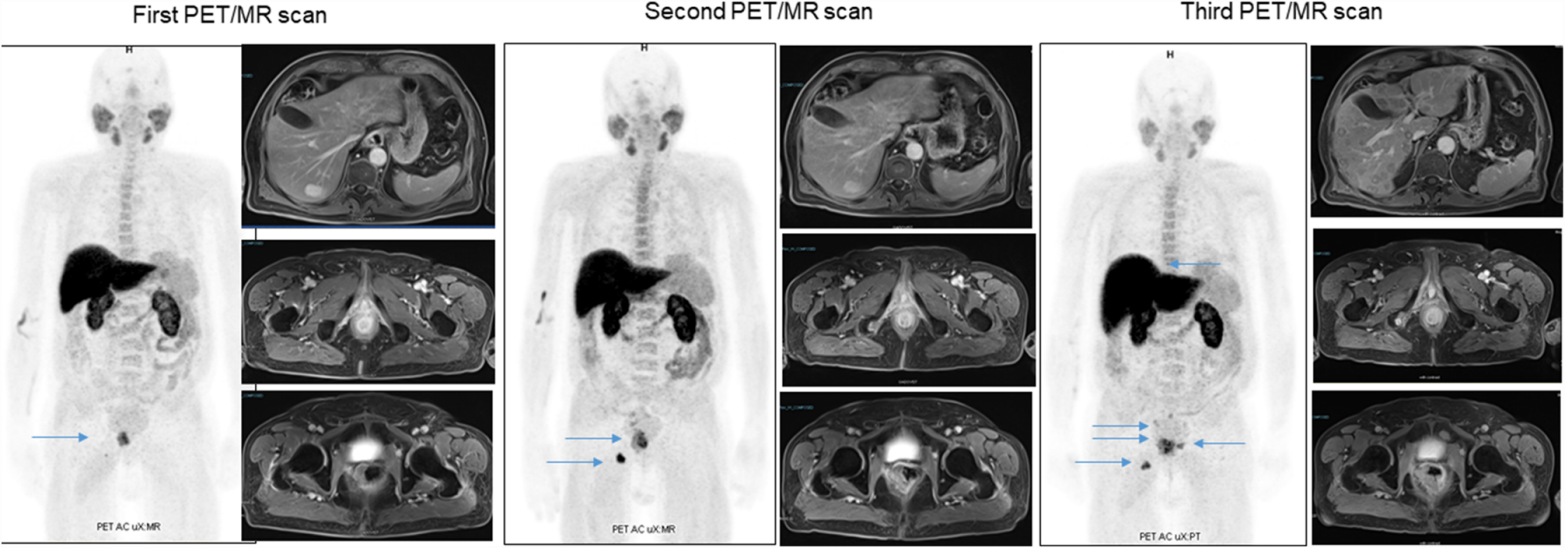
First, second and third [18F]F-choline PET/MR scan (maximum intensity projection on the left and transaxial fused images on the right, respectively), showing a para-rectal nodule (SUVmax 9, blue arrows) and bone (SUVmax 11.5 at the right ischium-pubic ramus) progressive disease, despite patient’s very low PSA values

**Fig. 7:**
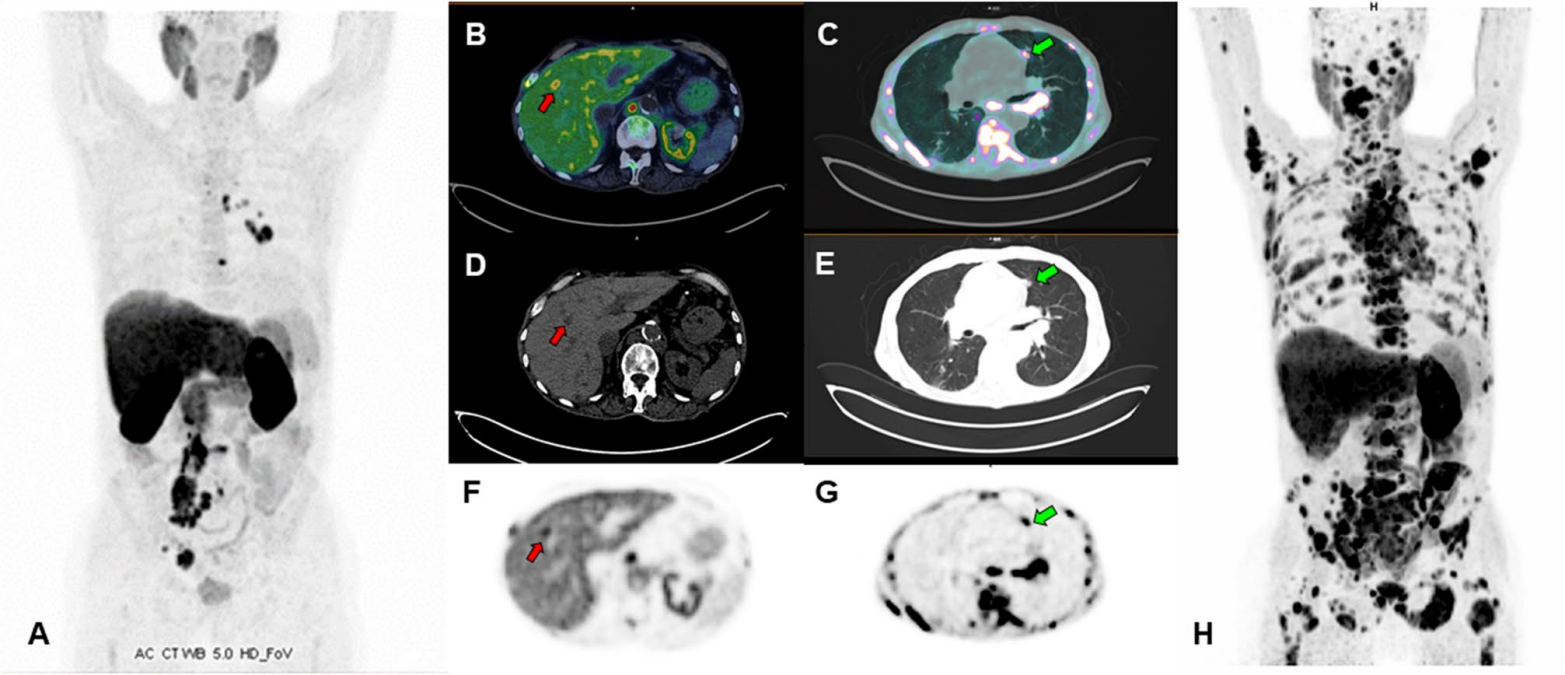
Baseline [18F]F-choline PET/CT scan (**A** maximum intensity projection) showed several skeletal and lymph nodes PCa metastasis uptake. Restaging [18F]F-choline PET/CT scan (**B**,**C** transaxial fused images; **D**,**E** CT images; **F**,**G** emissive images; H: maximum intensity projection), performed 12 months later, after starting treatment with metronomic cyclophosphamide, showed a multiple district disease progression, with lesions involving lymph nodes, bone, liver (red arrows, SUVmax 15) and both lungs (green arrows, SUVmax 5.1)

CASE 7. A 64-year-old man with PCa Gleason Score 9 (4 + 5), ISUP grade 4, was previously treated with radical prostatectomy and adjuvant radiation therapy. The serum PSA nadir (0.01 ng/ml) was achieved 3 months after surgery. Twenty-four months later, PSA serum value raised to 1.14 ng/ml, therefore a [18F]F-choline (Fig. 6) PET/CT was suggested. The images revealed recurrence of disease in lymph nodes. In the following years, the patient was treated with numerous lines of hormone therapy (LHRH-agonists, enzalutamide, and abiraterone acetate plus prednisone) and chemotherapy (docetaxel and cabazitaxel). After 9 years from the initial diagnosis, for a PSA elevation (4.19 ng/ml), a [18F]F-choline PET/CT was performed showing lymph node and bone progression of disease (Fig. 7A), therefore a new line of therapy with metronomic cyclophosphamide was started. After 6 months of treatment an important increase of serum PSA level (85.2 ng/ml) was found, and a new [18F]F-choline PET/CT scan revealed a marked lymph node, bone and lung progression of PCa (multiple subcentimetric, ranging between 5–8 mm (Fig. 7B–H)). The patient died shortly.

This case underlines the utility of [18F]F-choline PET/CT for revealing visceral metastases developed during systemic therapy. Although physiological uptake of [18F]F-choline in the liver can complicate the evaluation of hepatic metastases, particularly if small, lung metastases are pretty well evaluable, although being really small and uncharacterizable on conventional imaging. Despite being uncommon in PCa, visceral metastases usually appear in the late stage of disease and their presence is a recognized strong negative prognostic factor [26].

### Teaching points

- [18F]F-choline PET demonstrated to be a useful imaging modality in the assessment of response to systemic therapy in metastic PCa patients, irrespective of the stage of disease (either in hormone sensitive and in castrate resistant condition) and the kind of systemic treatment.
- [18F]F-choline PET should not be performed in close proximity to the start of ADT, or it might hesitate in false negative findings and down-staging of disease.
- In most cases PSA serum values and [18F]F-choline PET show a synchronous disease evolution after systemic therapy, although occasionally functional imaging could reveal a progressive disease not supported by the laboratory sample.
- Moreover, [18F]F-choline PET can be an useful tool for revealing the switch from CSPCa to CRPCa.
- [18F]F-choline PET could finally detect the presence of visceral metastasis, particularly in lungs.

## Conclusions

Despite the lack of prospective and large cohort trials, [18F]F-choline PET imaging is a promising tool to evaluate response to different systemic treatments in PCa. This pictorial essay should represent a starting point for future papers to deepen this issue. Future experiences should take in consideration also PSMA-ligands PET imaging, that may represent another useful imaging modality to fully understand patients’ response to therapy.

## References

[1] De Giorgi U, Caroli P, Scarpi E (2015). 18F-Fluorocholine PET/CT for early response assessment in patients with metastatic castration-resistant prostate cancer treated with enzalutamide. Eur J Nucl Med Mol Imaging.

[2] Glunde K, Bhujwalla ZM, Ronen SM (2011). Choline metabolism in malignant transformation. Nat Rev Cancer.

[3] Fuccio C, Castellucci P, Schiavina R (2012). Role of 11C-choline PET/CT in the re-staging of prostate cancer patients with biochemical relapse and negative results at bone scintigraphy. Eur J Radiol.

[4] Murphy RC, Kawashima A, Peller PJ (2012). The utility of 11C-choline PET/CT for imaging prostate cancer: a pictorial guide. Am J Roentgenol.

[5] Eisenhauer EA, Therasse P, Bogaerts J (2009). New response evaluation criteria in solid tumours: revised RECIST guideline (version 1.1). Eur J Cancer.

[6] Ceci F, Castellucci P, Graziani T (2016). 11C-Choline PET/CT in castration-resistant prostate cancer patients treated with docetaxel. Eur J Nucl Med Mol Imaging.

[7] Maines F, Caffo O, Donner D (2016). Serial 18F-choline-PET imaging in patients receiving enzalutamide for metastatic castration-resistant prostate cancer: response assessment and imaging biomarkers. Futur Oncol.

[8] Castello A, Castellani M, Florimonte L (2022). The role of radiomics in the era of immune checkpoint inhibitors: a new protagonist in the jungle of response criteria. J Clin Med.

[9] Uccelli L, Boschi A, Cittanti C (2021). 90 y/177 lu-dotatoc: from preclinical studies to application in humans. Pharmaceutics.

[10] Oprea-Lager DE, van Kanten MP, van Moorselaar RJA (2014). (2014) [18F]Fluoromethylcholine as a chemotherapy response read-out in prostate cancer cells. Mol Imaging Biol.

[11] Alongi P, Laudicella R, Lanzafame H (2022). PSMA and choline PET for the assessment of response to therapy and survival outcomes in prostate cancer patients: a systematic review from the literature. Cancers (Basel).

[12] de Giorgi U, Caroli P, Burgio SL (2014). Early outcome prediction on 18F-fluorocholine PET/CT in metastatic castration-resistant prostate cancer patients treated with abiraterone. Oncotarget.

[13] Schwarzenböck SM, Knieling A, Souvatzoglou M (2016). [11C]Choline PET/CT in therapy response assessment of a neoadjuvant therapy in locally advanced and high risk prostate cancer before radical prostatectomy. Oncotarget.

[14] Quaquarini E, D’Ambrosio D, Sottotetti F (2019). Prognostic value of 18 F-Fluorocholine PET parameters in metastatic castrate-resistant prostate cancer patients treated with docetaxel. Contrast Media Mol Imaging.

[15] Caffo O, Maines F, Donner D (2014). Impact of enzalutamide administration on primary prostate cancer volume: a metabolic evaluation by choline positron emission tomography in castration-resistant prostate cancer patients. Clin Genitourin Cancer.

[16] Urso L, Castello A, Rocca GC (2022). Role of PSMA-ligands imaging in renal cell carcinoma management: current status and future perspectives. J Cancer Res Clin Oncol.

[17] Martinez-Moller A, Souvatzoglou M, Delso G (2009). Tissue classification as a potential approach for attenuation correction in whole-body pet/mri: evaluation with PET/CT Data. J Nucl Med.

[18] Dost RJ, Glaudemans AWJM, Breeuwsma AJ, De Jong IJ (2013). Influence of androgen deprivation therapy on choline PET/CT in recurrent prostate cancer. Eur J Nucl Med Mol Imaging.

[19] Giovacchini G, Picchio M, Coradeschi E (2010). Predictive factors of [11C]choline PET/CT in patients with biochemical failure after radical prostatectomy. Eur J Nucl Med Mol Imaging.

[20] Hara T, Bansal A, DeGrado TR (2006). Effect of hypoxia on the uptake of [methyl-3H]choline, [1-14C] acetate and [18F]FDG in cultured prostate cancer cells. Nucl Med Biol.

[21] Kyriakopoulos CE, Chen YH, Carducci MA (2018). Chemohormonal therapy in metastatic hormone-sensitive prostate cancer: long-term survival analysis of the randomized phase III E3805 CHAARTED trial. J Clin Oncol.

[22] Clarke NW, Ali A, Ingleby FC (2019). Addition of docetaxel to hormonal therapy in low- and high-burden metastatic hormone sensitive prostate cancer: long-term survival results from the STAMPEDE trial. Ann Oncol.

[23] Poeppel TD, Handkiewicz-Junak D, Andreeff M (2017). EANM guideline for radionuclide therapy with radium-223 of metastatic castration-resistant prostate cancer. Eur J Nucl Med Mol Imaging.

[24] Schlack K, Krabbe L-M, Rahbar K (2021). ALP bouncing and LDH normalization in bone metastatic castration-resistant prostate cancer patients under therapy with Enzalutamide: an exploratory analysis. Transl Androl Urol.

[25] Jadvar H (2016). Is There utility for FDG PET in prosate cancer?. Semin Nucl Med.

[26] Pezaro CJ, Omlin A, Lorente D (2014). Visceral disease in castration-resistant prostate cancer. Eur Urol.

